# Rose geranium essential oil as a source of new and safe anti-inflammatory drugs

**DOI:** 10.3402/ljm.v8i0.22520

**Published:** 2013-10-07

**Authors:** Mohamed Nadjib Boukhatem, Abdelkrim Kameli, Mohamed Amine Ferhat, Fairouz Saidi, Maamar Mekarnia

**Affiliations:** 1 Laboratoire Eco-Physiologie Végétale, Département des Sciences Naturelles, Ecole Normale Supérieure de Kouba, Alger, Algeria; 2 Laboratoire Biotechnologies Végétales, Département de Biologie, Université Saad Dahleb de Blida, Algeria; 3 Laboratoire de Recherche sur les Produits Bioactifs et Valorisation de la Biomasse, Ecole Normale Supérieure de Kouba, Alger, Algeria; 4 Société Extral-Bio de Production des Huiles Essentielles et Cosmétiques Bio, route de Chiffa, Blida, Algeria

**Keywords:** essential oils, rose geranium, citronellol, anti-inflammatory effect, skin inflammation, histopathology, carrageenan, croton

## Abstract

**Background:**

Since the available anti-inflammatory drugs exert an extensive variety of side effects, the search for new anti-inflammatory agents has been a priority of pharmaceutical industries.

**Aims:**

The aim of the present study was to assess the anti-inflammatory activities of the essential oil of rose geranium (RGEO).

**Methods:**

The chemical composition of the RGEO was investigated by gas chromatography. The major components were citronellol (29.13%), geraniol (12.62%), and citronellyl formate (8.06%). In the carrageenan-induced paw edema, five different groups were established and RGEO was administered orally in three different doses.

**Results:**

RGEO (100 mg/kg) was able to significantly reduce the paw edema with a comparable effect to that observed with diclofenac, the positive control. In addition, RGEO showed a potent anti-inflammatory activity by topical treatment in the method of croton oil-induced ear edema. When the dose was 5 or 10 µl of RGEO per ear, the inflammation was reduced by 73 and 88%, respectively. This is the first report to demonstrate a significant anti-inflammatory activity of Algerian RGEO. In addition, histological analysis confirmed that RGEO inhibited the inflammatory responses in the skin.

**Conclusion:**

Our results indicate that RGEO may have significant potential for the development of novel anti-inflammatory drugs with improved safety profile.

The World Health Organization projected that 80% of people in emerging nations rely on medicinal plants for primary health care needs. The cost of acquiring synthetic drugs, their insufficient supplies, the side effects associated with their uses, and the belief that plants hold the cure for many disease conditions (including inflammatory disorders) has led to a reawakening of interest in the utilization of plants and plant extracts in recent years. There is a need to expand scientific investigation into medicinal plants especially those claiming to have beneficial effects in serious illnesses (1, 2).

Commercially available anti-inflammatory drugs exert a wide range of side effects and are either too potent or too weak. Consequently, the search for new anti-inflammatory compounds has been a priority for the pharmaceutical industry. Medicinal plants continue to be an important source of new chemical substances with potential therapeutic effects (3). Numerous natural products have been tested in various animal models for the development of new anti-inflammatory agents. Plant essential oils (EO) are used as folk medicine against various kinds of inflammatory diseases. Some of them have also been scientifically shown to possess medicinal activities, including anti-inflammatory activities, in experimental systems *in vitro* and *in vivo* (4–6).

In Algeria, the use of medicinal plants as anti-inflammatory drugs is a common practice, although in most cases the active principles of the plants are unknown. Therefore, the study of plant species should still be seen as a logical research strategy, in search for new anti-inflammatory drugs. The variety of climatic and geographic conditions in Algeria provides a rich source of vegetation, comprising many species of plants (7). Among these plants, rose geranium, which belongs to the Geraniaceae family, has been widely used for its antibacterial and antifungal actions. The RGEO is extracted from the leaves and stems of the plant by steam distillation. The RGEO is composed of various chemical constituents such as linalool, citronellol, geraniol, and their esters (8, 9). Rose geranium is one of the most fragrant species and its EO is used in the perfumery and cosmetics industry. It is also used as a flavoring agent and as a spice. Further, the RGEO is non-toxic, non-irritant, generally non-sensitizing, and it is not known to cause any other side effects. The therapeutic properties of RGEO include being an antidepressant, antiseptic and wound-healing (vulnerary). RGEO may also be one of the best oils for diverse dermatological problems such as oily or congested skin, eczema, and dermatitis (10, 11).

Presently, there are no published scientific data to validate the popular claims of anti-inflammatory activity of the plant. The purpose of the present study was to evaluate the anti-inflammatory activities of RGEO using the carrageenan-induced paw edema and croton oil-induced ear edema tests. In addition, we describe the identification of the various constituents of RGEO by gas chromatography − mass spectrometry (GC–MS).

## Material and methods

### Plant material and EO extraction

Rose-scented geranium was cultivated in the aromatic garden of ‘Extral-Bio’ Company (Blida city, Algeria). The aerial parts of the plant were collected in September 2012. Identification of the plant was confirmed by the National Institute of Agronomy (Algiers, Algeria).

EO was obtained by steam distillation of fresh plant material (Extral-Bio Company) in a stainless steel distillation apparatus (alembic) for 3 h. The procedure consists of passing water vapor at low pressure through a tank containing the aromatic plant parts. The steam captures the oil trapped in micro-pockets within the plant tissue. The steam then passes through a cold-water refrigerated serpentine to be condensed into liquid. Upon exit, the collected liquid is a mixture of oil and floral water, which are easily separated using a Florentine vase. The RGEO thus obtained was dried over anhydrous sodium sulfate, filtered, and stored at +4°C until tested.

### GC–MS analyses

GC analyses were performed using a Hewlett-Packard (HP, Palo Alto, CA, USA) gas chromatograph equipped with a flame ionization detector and HP5-MS capillary column (30 m, 0.32 mm, 0.25 µm film thickness). The oven temperature was programmed isothermally for 8 min at 45°C and then 45–240°C at 2°C/min for 15 min. Injector and detector temperatures were 250 and 280°C, respectively. Carrier gas was nitrogen at a flow rate of 1.2 ml/min in split mode 1:70 with an injection volume of 1 µl. The composition of RGEO was computed by the normalization method from the GC peak areas.

GC–MS analyses were performed using a Hewlett-Packard GC system interfaced with a mass spectrometer equipped with an HP5-MS capillary column (30 m, 0.32 mm, 0.25 µm film thicknesses). For GC–MS detection, electron ionization with ionization energy of 70 eV was used. Helium was the carrier gas at a flow rate of 1.2 ml/min with an injection volume of 1 µl. Injector and detector temperatures were set at 250 and 280°C, respectively.

Identification of the components of rose geranium volatile oil were made by matching their recorded mass spectra with the mass spectra data bank (Wiley 7N and NIST 2002 libraries) and by comparing their retention indices (RIs) relative to a series of hydrocarbons (C7–C28) with literature values (12).

### Experimental animals

Carrageenan-induced paw edema and croton oil-induced ear edema was carried out on male Swiss mice (25 − 30 g). Male animals were purchased from the laboratory of toxicology of Antibiotical Saidal Company (Medea, Algeria) and were housed in groups of six per standard cage, on a 12 h light/dark cycle with free access to food and water. They were acclimatized to laboratory conditions for at least 1 week before testing. The food was withdrawn on the day before the experiment, but free access to water was allowed. A minimum of six animals was used in each group.

### Determination of median lethal dose (LD_50_)

LD_50_ of the RGEO was estimated in mice by using the method of Hilan et al. (13). In a preliminary test, animals in groups of three received 10, 100, or 1,000 mg/kg of RGEO suspended in the vehicle (1% v/v Tween 80). Animals were observed for 24 h for signs of toxicity and number of deaths. The LD_50_ was calculated as the geometric mean of the dose that resulted in 100% mortality and that which caused no deaths.

### *In vivo* anti-inflammatory activity assay

#### Carrageenan-induced paw edema in mice

The anti-inflammatory activity was evaluated by the carrageenan-induced paw edema test (14). Paw edema was induced by injecting 0.1 ml of the carrageenan 1% suspension in isotonic saline (w/v) into the sub-plantar region of the left hind paw of the mouse.

RGEO doses of 100, 200, or 400 mg/kg and vehicle (0.2% Tween 80 in 0.9% NaCl) were administrated (0.5 ml per animal) orally (per os) 30 min before injection of the edematogenic agent to different groups of mice for each treatment (*n=*6 per group). Diclofenac sodium dissolved in 0.9% NaCl (50 mg/kg, oral) was used as a reference drug. Paw thickness was measured before the application of the inflammatory substance and every 30 min for 4 h after induction of inflammation. The difference in footpad thickness was measured by a gauge calliper (Facom, Paris, France).

Mean values of treated groups were compared with those of control group (vehicle) and analyzed statistically. The data obtained for the various groups are reported as means±standard deviation (SD) and expressed in mm. The percentage inhibition of the inflammatory reaction was determined for each animal by comparison to the controls and calculated by the formula:

$$I(\%)=\left[1-\frac{\Delta {\left(\mathrm{PV}\right)}_{t}}{\Delta {\left(\mathrm{PV}\right)}_{c}}\right]\times 100$$

where I (%)=percent inhibition of edema, (▵PV)*t=*the change in paw volume in the treated mice, and (▵PV)*c=*the change in paw volume in the control mice.

#### Croton oil-induced ear edema in mice

To estimate the anti-inflammatory activity of RGEO in vivo, we used the acute inflammation model of croton oil-induced mouse ear edema according to the method described by Sosa et al. (15). An acetone solution of croton oil (100 µg/15 µl) was carefully applied to the inner surface of the left ear of each mouse. The right ear remained untreated. Vehicle (2 ml/kg) and different doses of the RGEO (200 or 400 µl/kg) were applied topically to the left ear about 30 min before the croton oil treatment. As a reference, we applied 1 mg/ear of the non-steroidal anti-inflammatory drug, diclofenac sodium 1% gel (Voltaren Emulgel, Novartis, France). For evaluation of the activity, two different ways were followed:

1. The thickness of each ear was measured with a gauge calliper 4 h after induction of inflammation. The edema was expressed as the difference between the right and left ears.
2. Four hours after induction of inflammation, the mice were sacrificed and a tissue sample (a plug 5 mm in diameter) was removed with a Punch Biopsy (LCH Medical products, Paris, France) from both treated (left) and untreated (right) ears. The percentage inhibition of the inflammation was determined for each animal by comparison to the controls and calculated by the following formula:

$$I(\%)=\left[1-\frac{\Delta {\left(\mathrm{WT}\right)}_{t}}{\Delta {\left(\mathrm{WT}\right)}_{c}}\right]\times 100$$

where I (%)=percent inhibition of edema, (▵WT)*t=*the change in weight of ear tissue in the treated mice, and (▵WT)*c=*the change in weight of ear tissue in the control mice.

#### Histopathological examination of mouse ear tissue

For morphological assessment of cutaneous inflammation, biopsies from control and treated ears of mice in each treatment group were collected at the end of the experiment. Samples were fixed in 4% formaldehyde and decalcified. Fixed tissues were serially sliced at a thickness of 5.0 µm using a microtome (Leica, Nussloch, Germany). The sections were stained with Harry's Hematoxylin-Eosin. The tissues were examined by a light microscope (Olympus) without blinding and graded for edema as mild (+), moderate (+ + ), or severe (+ + +). The tissue samples were also examined for epidermal hyperplasia and for inflammatory cell infiltration (mononuclear and/or polymorphonuclear cells) in the dermis inflammation phase.

### Statistical analysis

Results of the paw edema of the mice are reported as mean±SD. Comparison between groups was made by one-way analysis of variance (ANOVA) followed by Tukey's *post hoc* multiple comparison test. Differences with *P*<0.05 were considered statistically significant. Statistical data analysis was determined by probit analysis using XLStats 2013 Pros statistical software (Addinsoft, Paris, France).

## Results and discussion

### Chemical composition of volatile oil

The EO was obtained by steam distillation in a stainless steel alembic from fresh aerial part of rose-scented geranium. The rose geranium oil obtained is a yellowish-green liquid. It has a strong lemon-rose odor. The EO was obtained with a yield of 0.15% (v/w). In a recent study (16), it has been reported that a higher yield is obtained during spring/summer (0.1%) than during autumn/winter, with an average yield of 0.06%.

The volatile oil was analyzed by GC-MS. Qualitative and quantitative studies of the oil volatile profiles are listed in Table 1 in order of their retention indices. In total, 20 compounds representing 83.5% of the EO were identified. Citronellol (29.1%), geraniol (12.6%), citronellyl formate (8.1%), geranyl tiglate (7.1%), and linalool (4.5%) were the major compounds in the oil, with minor quantities of geranyl butyrate (2.0%) and geranyl acetate (1.6%). Other constituents were found in smaller amounts (<2%). The rose geranium oil consisted mainly of oxygenated monoterpenes (76.9%) and oxygenated sesquiterpenes (3.3%).

**Table 1:** Chemical profile of rose geranium essential oil extracted by steam distillation

No.	Compounds^a^	RI	Content %
1	*α*-Pinene	926	0.85
2	Linalool	1,125	4.52
3	*cis*-Rose oxide	1,129	0.92
4	*trans*-Rose oxide	1,131	0.36
5	Menthone	1,156	4.21
6	Citronellol	1,167	29.13
7	Geraniol	1,271	12.62
8	Citronellyl formate	1,275	8.06
9	Geranyl formate	1,300	3.46
10	Citronellyl acetate	1,342	0.43
11	*α*-Copaene	1,356	1.13
12	Geranyl acetate	1,366	1.58
13	Caryophellene	1,391	1.76
14	Citronellyl propanoate	1,444	0.66
15	*α*-Agarofuran	1,545	0.4
16	Geranyl *N*-butyrate	1,562	2.02
17	Phenylethyl tiglate	1,584	1.26
18	10-epi-gamma-Eudesmol	1,619	3.31
19	Citronellyl tiglate	1,667	0.48
20	Geranyl tiglate	1,700	7.14
	Total identified		83.45
	Oxygenated monoterpenes		76.85
	Monoterpene hydrocarbons		1.13
	Oxygenated sesquiterpenes		3.31
	Sesquiterpene hydrocarbons		2.16

RIs (retention indices) were calculated on the HP-5 MS column relative to C7-C28 n-alkanes.

a Compounds listed in order of elution from an HP-5 MS column.

The data presented here are consistent with previous reports (9, 17), which demonstrated that rose geranium oils are characterized by citronellol (22.0–32.9%) as the most important component. However, our results diverge from those published by other studies (18). Generally, the observed differences in chemical composition of rose geranium oils, when compared with those reported in previous studies could be due to a number of factors, including differences in climatic conditions and geographical locations, season at the time of collection, and fertilization (17, 19). Previous reports revealed that although the chemical composition of the RGEO differed owing to the geographical origin, compounds such as alcohols, ketones, esters, and mainly aldehydes have consistently been recorded (8, 17).

### Determination of median lethal dose (LD_50_)

The RGEO did not cause any mortality in the mice in doses up to 1,000 mg/kg. Therefore, we suggest that oral LD_50_ of the tested volatile oil > 1,000 mg/kg. Thus, this oil can be considered as highly safe. Geranium oils were granted GRAS status (Generally Recognized As Safe) and approved by the US Food and Drug Administration (FDA) for food use (8).

### Anti-inflammatory activity of EO

#### Carrageenan-induced paw edema in mice

The anti-inflammatory effect of EO was evaluated in carrageenan-induced paw edema in mice, an animal model widely employed to assess the anti-edematogenic effect of natural products. Carrageenan is commonly used as a phlogistic (inflammation-inducing) agent. The resulting signs and symptoms of inflammation can be measured as an increase in paw thickness due to the edema.

The anti-inflammatory effect of the EO (100 − 400 mg/kg) was evaluated in the paw edema model (*n=*6 per group). As shown in Table 2, the oral administration of EO at doses of 100, 200 and 400 mg/kg resulted in 30, 38 and 73% reduction in paw edema, respectively. Furthermore, the inhibition of paw edema resulting from a 100-mg/kg EO dose was not significantly different from that of diclofenac (50 mg/kg) (73.1% vs. 80.8%, *P*>0.05). This is the first demonstration that oral administration of RGEO produces significant anti-inflammatory effects.

**Table 2:** Effect of rose geranium essential oil on carrageenan-induced paw edema in mice (*n*=6)

Treatment	Dose (mg/kg)	Thickness of the left hind paw (mm), mean±SD	Inhibition of paw edema (%)
Negative control	20	3.1±0.15^d^	–
RGEO	400	3±0.05^c^	30.76
RGEO	200	2.88±0.11^b^	38.46
RGEO	100	2.81±0.17^a^	73.07
Diclofenac	50	2.76±0.12^a^	80.76

Groups if mice were pretreated with vehicle (control group, 20 mg/kg, p.o., *n*=6) diclofenac (50 mg/kg) or rose geranium essential oil at doses of 100, 200 and 400 mg/Kg (p.o., *n*=6/group) 30 min before carrageenan-induced paw edema. Means within the same column followed by the same small letter are not significantly different (*P*>0.05) according to ANOVA one-way analysis followed by Tukey's *post hoc* multiple comparison test.

This evidence allows us to suggest that the anti-inflammatory actions of RGEO are related to the inhibition of one or more intracellular signaling pathways involved in the effects of several inflammatory mediators. A study carried out by Abe et al. (20) showed that the EO of geranium suppressed the adherence response of human neutrophils *in vitro*, and the intra-peritoneal administration of this oil to mice lowered induced neutrophil recruitment into the peritoneal cavity.

Results from an investigation carried out on a number of EOs established that a single intra-peritoneal injection of RGEO clearly suppressed the carrageenan-induced foot paw edema, and repeated administration of the oil suppressed collagen-induced arthritis. These results revealed that RGEO suppressed both acute and chronic inflammatory responses in mice (21, 22).

Attempts have been made to identify the component(s) responsible for such bioactivities (23). Some plant constituents, particularly alcohol terpenoids (geraniol and citronellol), have been reported to be useful in the management of inflammatory processes (23–26). Our results are in agreement with those reported in the literature for other EOs rich in monoterpenic alcohol and showing a very strong anti-inflammatory effect. Further, the major constituents of the oil, namely citronellol, geraniol, and linalool, were previously shown to possess anti-inflammatory activities (20, 27). It has been reported (24) that RGEO might be beneficial in the prevention/treatment of neurodegenerative diseases in which inflammation is part of the pathophysiology.

#### Croton oil-induced ear edema in mice

Since we found that RGEO has anti-inflammatory activity in carrageenan-induced edema, we evaluated its activity further in croton oil-induced ear edema to assess the potential anti-inflammatory effect of topically applied RGEO *in vivo*. The results showed dose-dependent reduction of ear edema (Table 3). RGEO at doses of 200 or 400 µl/kg applied topically produced 73 and 88% inhibition of ear edema, respectively. Diclofenac sodium (40 mg/kg) produced 85% inhibition of croton oil-induced inflammation, and this effect was not statistically different from that observed with the maximum dose of RGEO. To the best of our knowledge, this is the first report to demonstrate that Algerian RGEO possesses significant anti-inflammatory activity.

**Table 3:** Topical application of rose geranium essential oil prevents croton oil-induced ear edema in mice

Treatment	Dose (µl/kg)	Weight edema (mg) mean±SD	Inhibition (%)
Negative Control	50	23.75±7.67^c^	–
RGEO	200	11.25±3.30^b^	73.52
RGEO	400	8.75±2.21^a^	88.23
Diclofenac	40	9.25±3.09^a^	85.29

Data are presented as mean (mm)±standard deviation (SD) (*n*=6 per group).

Means within the same column followed by the same small letter are not significantly different (*P*>0.05) according to ANOVA one-way analysis followed by Tukey's *post hoc* multiple comparison test.

Increased skin thickness is often the first hallmark of skin irritation and local inflammation. This parameter is an indicator of a number of processes that occur during skin inflammation, including increased vascular permeability, edema and swelling within the dermis, and proliferation of epidermal keratinocytes. In our study, treatment with EO (200 or 400 µl/kg) caused significant decreases in edematous ear thickness of 0.19 and 0.15 mm, respectively.

In accordance with our results, Abe et al. (20) reported that cutaneous application of EOs, especially RGEO, can suppress inflammatory symptoms observed as reductions in neutrophil accumulation and edema. More recently, the efficacy of RGEO and geraniol was tested against vaginal candidiasis as well as vaginal inflammation and *Candida* growth form. The vaginal application of RGEO successfully suppressed *Candida* cell growth in the vagina and its local inflammation when combined with vaginal washing, demonstrating the protective effect of RGEO and its main monoterpene against vaginal inflammation in mice (25).

Topical application of certain EOs, including RGEO, is popular in the practice of aromatherapy and body massage. Several of these oils are used to treat inflammatory conditions with neutrophil accumulation: rheumatoid arthritis, aphthous stomatitis, and bacterial or fungal infections (20).

### Histopathology analyses of ear tissue

**Fig. 1:**
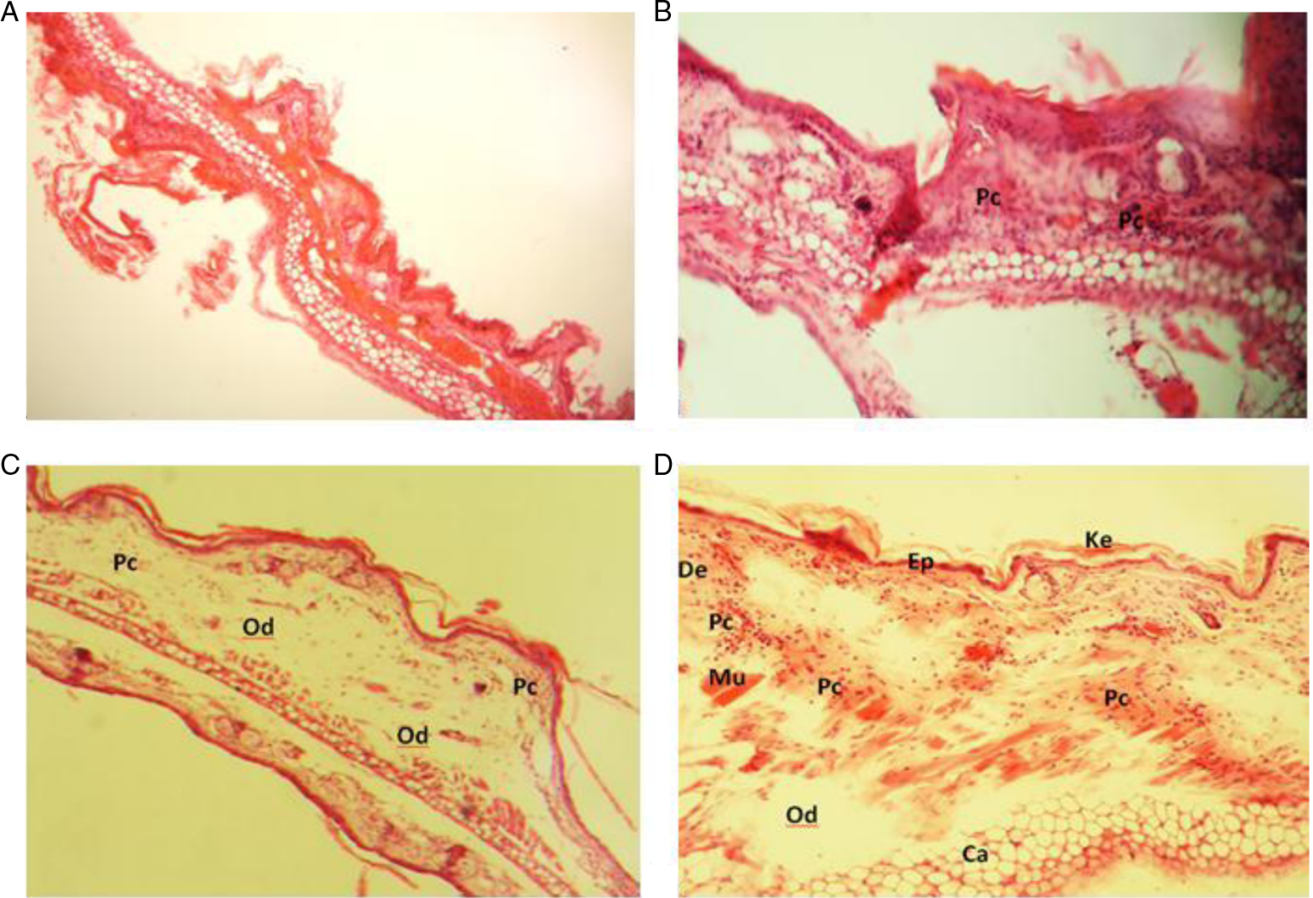
Histopathology sections of mouse ear biopsies representing keratin, epidermal, dermal, and cartilage layers (magnification xl00). Harry's hematoxylin-eosin stained sections were scored as mild (+), moderate (+ + ), and severe (+ + +) for edema and substantial inflammatory mononuclear and/or polymorphonuclear cell infiltration in the dermis inflammation phase. (A) no treatment; (B) rose geranium essential oil treatment: edema (−); inflammatory cell infiltration (+), inflammation phase (±). (C) and (D) croton oil: edema (+ + +); inflammatory cell infiltration (+ + ), inflammation phase (+ + ). Ke: keratin; Ep: epidermal layer; De: dermal layer; Mu: muscle; Ca: cartilage layer; Od: edema; Pc: polymorphonuclear cell infiltration.

We examined H&E-stained ear sections from croton oil treated animals. Local application of croton oil resulted in a marked increase in ear thickness with clear evidence of epidermal hyperplasia, edema, and substantial inflammatory cell infiltration in the dermis with associated connective tissue disturbance (Fig. 1C and D). Based on the histological assessment, RGEO treatment reduced edematous ear thickness and associated pathological indicators to an extent comparable to the positive control, diclofenac (Fig. 1B). The results of the histopathology analysis were similar in carrageenan and croton oil-induced edema methods. These results directly illustrate the effects of RGEO within the target tissue, providing further evidence that RGEO ameliorates croton oil-induced contact dermatitis. Our results are in agreement with a study by Al-Reza et al. (28), whose histological analysis revealed that Jujuba fruit oil inhibited the inflammatory responses of skin inflammation.

## Conclusions

RGEO is used as an antibacterial and antifungal agent in folk medicine as well as a food preservative. The results of our present study together with those of other researchers give strong impetus to the consideration of RGEO as a potentially useful anti-inflammatory agent both for the prevention and treatment of acute or chronic inflammatory skin diseases. In addition, study of the major chemical constituents of RGEO might accelerate the development of new, effective, and safe anti-inflammatory drugs.
